# Supplementary material for: Humidification Practices of Extremely Preterm Neonates: A Clinical Survey
Source: Healthcare (Basel). 2022 Jul 31;10(8):1437. doi: 10.3390/healthcare10081437 (PMC9408417; doi:10.3390/healthcare10081437)
Supplement: Supplementary file 1 [file healthcare-10-01437-s001.zip › healthcare-1792804-supplementary-Document S1.pdf]

Humidification Practices and Guidelines for Extremely Premature Neonates

Dear Practitioner,

We are currently reviewing our humidification practices for extremely preterm neonates and would appreciate your completion of this short survey with a description of the current practices of your neonatal intensive care unit (NICU). This survey is a research project aimed at determining the variability in humidification practices employed in NICUs – both nationally and internationally. In addition, the results of the survey will be used to develop a consensus guideline for humidification practices for the NICU at Golisano Children's Hospital/University of Rochester School of Medicine (GCH/URSMD). The results of this survey may be published.

The survey should take no longer than 5 to 10 minutes, and has been approved by the University of Rochester Institutional Review Board. Responses will be anonymous and no individuals will be identified in any published material. By completing this survey you have indicated consent for the survey and the use of your responses. If you are unsure of your unit's practice, please forward the link to a member of your staff who may be able to answer the survey. The group conducting this survey are:

Ms. Nina Rizk - Medical Student, University of Rochester School of Medicine and Dentistry

Ms. Lauren Bopp - Nurse, Neonatal Intensive Care Unit, Golisano Children's Hospital at URMC

Ms. Karen Paul - Nurse Manager, Neonatal Intensive Care Unit, Golisano Children's Hospital at URMC

Ms. Rachel Jones - Human Subject Research Coordinator, Division of Neonatology at URMC

Dr. Carl D'Angio - Chief, Division of Neonatology, University of Rochester, GCH/ URMC

Dr. Alison Kent - Director of Neonatal Clinical Research, Division of Neonatology, University of Rochester, GCH/URMC, Australian National University, College of Health and Medicine

1. Please indicate your country of practice.
   - [ ] United States
   - [ ] Canada
   - [ ] Australia
   - [ ] New Zealand
   - [ ] India
   - [ ] Other (Please Specify) _______________

2. Please indicate your professional status.
   - [ ] MD
   - [ ] NP
   - [ ] RN
   - [ ] APP
   - [ ] Other (Please Specify) _______________

3. Please provide the name of your institution.

   ____

4. Does your institution have a guideline for isolette humidification?
   - ☐ Yes
   - ☐ No
   - ☐ Don't Know

5. How often do you change out the isolettes with humidification?
   - ☐ Every 1 Week
   - ☐ Every 2 Weeks
   - ☐ Other (Please Specify) _______________

6. How often do you change out the isolettes without humidification?
   - ☐ Every 1 Week
   - ☐ Every 2 Weeks
   - ☐ Other (Please Specify) _______________

7. Do you routinely administer candida prophylaxis treatment during humidification?
   - ☐ Yes
   - ☐ No
   - ☐ Other (Please Specify) _______________

8. At what gestational age do you begin humidification?
   - ☐ </= 28 Weeks
   - ☐ </= 27 Weeks
   - ☐ </= 26 Weeks
   - ☐ </= 25 Weeks
   - ☐ Other (Please Specify) _______________

9. At what percentage do you begin humidification?
   - ☐ 90% or Higher
   - ☐ 80% to 89%
   - ☐ 70% to 79%
   - ☐ 60% to 68%
   - ☐ 50% to 59%
   - ☐ 49% or Lower

- [ ] Other (Please Specify) _______________

10. At what post-natal age do you wean humidification?
    - [ ] 1 Day
    - [ ] 3 Days
    - [ ] 5 Days
    - [ ] 7 Days
    - [ ] Not Applicable
    - [ ] Other (Please Specify) _______________

11. At what percentage increments do you wean humidification?
    - [ ] 5%
    - [ ] 10%
    - [ ] Not Applicable
    - [ ] Other (Please Specify) _______________

12. At what post-natal age do you end humidification?
    - [ ] 1 Day
    - [ ] 3 Days
    - [ ] 5 Days
    - [ ] 7 Days
    - [ ] Other (Please Specify) _______________

13. At what percentage do you cease humidification?
    - [ ] 50% or Higher
    - [ ] 40% to 49%
    - [ ] 39 % or Lower

14. At what rate do you administer intravenous fluids during the first 24hrs for neonates 25 weeks and younger gestation?
    - [ ] 70mL/kg/day
    - [ ] 80mL/kg/day
    - [ ] 90mL/kg/day
    - [ ] 100mL/kg/day
    - [ ] Other (Please Specify) _______________

15. At what rate do you administer intravenous fluids during the first 24hrs for neonates 26 to 28 weeks gestation?
    - [ ] 70mL/kg/day
    - [ ] 80mL/kg/day
    - [ ] 90mL/kg/day
    - [ ] 100mL/kg/day
    - [ ] Other (Please Specify) _______________
